# Monocyte adhesive hyaluronan matrix induced by hyperglycemia in diabetic lung injuries

**DOI:** 10.1016/j.jbc.2023.104995

**Published:** 2023-06-30

**Authors:** Andrew Jun Wang, Juan Ren, Aimin Wang, Vincent C. Hascall

**Affiliations:** Department of Biomedical Engineering, Cleveland Clinic, Cleveland, Ohio, USA

**Keywords:** diabetic lung injury, smooth muscle cells, heparin, inflammation, hyaluronan matrix, hyperglycemia, mesangial cells, extracellular matrix

## Abstract

Infiltrated pre-inflammatory monocytes and macrophages have important roles in the induction of diabetic lung injuries, but the mechanism mediating their infiltration is still unclear. Here, we showed that airway smooth muscle cells (SMCs) activated monocyte adhesion in response to hyperglycemic glucose (25.6 mM) by significantly increasing hyaluronan (HA) in the cell matrix, with concurrent 2- to 4-fold increases in adhesion of U937 monocytic-leukemic cells. The HA-based structures were attributed directly to the high-glucose and not to increased extracellular osmolality, and they required growth stimulation of SMCs by serum. Treatment of SMCs with heparin in high-glucose induces synthesis of a much larger HA matrix, consistent with our observations in the glomerular SMCs. Further, we observed increases in tumor necrosis factor-stimulated gene-6 (TSG-6) expression in high-glucose and high-glucose plus heparin cultures, and the heavy chain (HC)-modified HA structures existed on the monocyte-adhesive cable structures in high-glucose and in high-glucose plus heparin-treated SMC cultures. Interestingly, these HC-modified HA structures were unevenly distributed along the HA cables. Further, the *in vitro* assay with recombinant human TSG-6 and the HA14 oligo showed that heparin has no inhibitory activity on the TSG-6-induced HC-transfer to HA, consistent with the results from SMC cultures. These results support the hypothesis that hyperglycemia in airway smooth muscle induces the synthesis of a HA matrix that recruits inflammatory cells and establishes a chronic inflammatory process and fibrosis that lead to diabetic lung injuries.

Diabetic lung dysfunction involves chronic inflammation, airway epithelial cell dysfunction, airway smooth muscle cell (SMC) hypertrophy, and impeded resolution, but the mechanisms are largely unknown (1, 2, 3, 4, 5, 6, 7, 8). Many diabetic complications share a common association with extensive and chronic inflammation due to the infiltration of activated leukocytes that originate from the bone marrow (BM) (9). Diabetes is the increased risk factor for pulmonary diseases such as asthma that are associated with hyperglycemia and result in extensive and chronic inflammation (1, 2, 3, 7, 10, 11). Previous studies have suggested that the interplay between various events at both the cellular and molecular levels results in the progression of diabetic lung dysfunction, including: (1) increases in monocyte/macrophage pro-inflammatory activation (9, 12, 13, 14, 15, 16, 17) and lung inflammation (2, 6, 18, 19); (2) increases in IL-17 and TGF-β production (20); (3) increases in airway hyper-responsiveness (AHR) and fibrosis (2, 4, 5, 6); and (4) decreased resolution responses (13). Airway inflammation and fibrosis, a key feature in the lung injuries of diabetes and obesity, is preceded by a phenotypic activation and proliferation of the airway smooth muscle cells and by a prominent infiltration of monocytes and macrophages, followed by a prominent IL-17 production (12, 13, 14, 15, 18, 19, 20). The activated pro-inflammatory monocytes and macrophages, prominently identified in diabetes (12), appear to have key roles to induce inflammatory processes in the lung that are not yet clearly understood (2, 3, 6, 14). The molecular mechanisms underlying airway infiltration and activation by monocytes in diabetic lung injuries are still unclear.

Hyaluronan (HA) is a linear glycosaminoglycan composed of repeating disaccharide units of *N*-acetylglucosamine and D-glucuronic acid with alternating β-1,4 and β-1,3 glycosidic bonds. It is a major, ubiquitous component of extracellular matrices. The number of repeat disaccharides in a completed HA molecule can reach 20,000 or more, a molecular mass of >8 million Da, and a length of >20 μm. The HA biosynthesis is tightly regulated by cellular metabolic status (21). Our previous studies show that hyperglycemic dividing cells activate HA synthases in intracellular membranes entering the S phase (22). This inserts the large, polyanionic HA *intracellularly* into ER, Golgi, and transport vesicles. This induces ER stress and autophagy responses, and extrusion of an *extracellular* monocyte-adhesive HA matrix after division, which provides compelling evidence for a causal link between increased glomerular HA matrix and monocyte/macrophage accumulation (22, 23, 24, 25).

Further, the HA matrix can be covalently modified by heavy chains (HCs) to form the HC–HA complexes. This reaction is catalyzed by tumor necrosis factor-stimulated gene-6 (TSG-6) in which the HCs are transferred from inter alpha *trypsin* inhibitor (IαI) and pre-IαI to HA molecules (26). IαI and pre-IαI are serum macromolecules synthesized by hepatocytes in the liver (27). IαI contains three polypeptides: bikunin (16 kDa) and two HCs (∼83 kDa each) (28), and the two HCs (HC1 and HC2) are covalently attached to the single chondroitin sulfate glycosaminoglycan of bikunin, while pre-IαI consists of a single HC (HC3) attached to the chondroitin sulfate chain. TSG-6 is a 35-kDa protein that binds to HA *via* its link module and has also been shown to form a complex with both HCs of IαI (29, 30). It is known to catalyze the transfer of HCs from their ester linkage to 6-OH of chondroitin sulfate GalNAc residues on IαI and pre-IαI to the 6-OH of GlcNAc residues in HA (31, 32, 33). The HC–HA complexes can be found in various cells and tissues that have important roles in mediating both physiological and pathological processes (34, 35, 36).

Understanding these cellular and molecular events can provide significant insights into the mechanisms controlling cellular responses to hyperglycemia that initiate the progression of diabetic lung injuries. We propose that the activation of pro-inflammatory monocytes/macrophages (Mpi) and their interaction with the pathological HA matrix initiates an inflammatory process that is responsible for subsequent matrix expansion and also as a source of more TGF-β (37). The increased TGF-β may inhibit the early proliferative phase by arresting the airway SMCs at the G1 phase of the cell cycle, thereby accelerating their subsequent hypertrophy and matrix synthesis.

Heparin is a highly sulfated, hence a highly polyanionic, glycosaminoglycan with a repeating disaccharide that contains a hexuronic acid (either glucuronic acid or iduronic acid) and glucosamine (either *N*-acetylated or *N*-sulfated). It is synthesized as a proteoglycan (serglycin) that is found in mast cells (38). Low concentrations (K_d_ ∼ 20 nM) of heparin prevent intracellular HA synthesis and reprogram the cells to synthesize a monocyte-adhesive extracellular HA matrix after division (22, 39). However, the molecular and cellular mechanism(s) underlying the beneficial roles of heparin in diabetic lung injuries and the regulatory roles of heparin in the formation of an extracellular HA matrix are still unknown.

This study determines the formation of the monocyte adhesive HA matrix by SMCs in hyperglycemic glucose and to what extent the heparin and 4-Methylumbelliferyl-β-D-xyloside (4MU-xyl) regulate these high glucose-induced responses in SMCs. Experiments described in this report indicate that: (1) cultures of airway smooth muscle cells exposed to high glucose can produce a HA matrix that promotes monocyte adhesion; (2) this process depends on the growth state and activation of the SMCs; (3) the HA matrix was modified by HC transfer to HA from inter-α*-trypsin inhibitor* (IαI) or pre-IαI induced by tumor necrosis factor-stimulated gene-6 (TSG-6); and (4) the heparin and 4MU-xyl regulate these high glucose-induced responses in SMCs. These results support the hypothesis that HA structures in the airway smooth muscle, produced in response to hyperglycemia, bind to specific receptors on the monocyte surface, thereby promoting monocyte and macrophage adhesion to the smooth muscle matrix in the early stages of diabetic lung injuries.

## Results

### Glucose-induced HA synthesis and monocyte adhesion in SMCs

**Figure 1:**
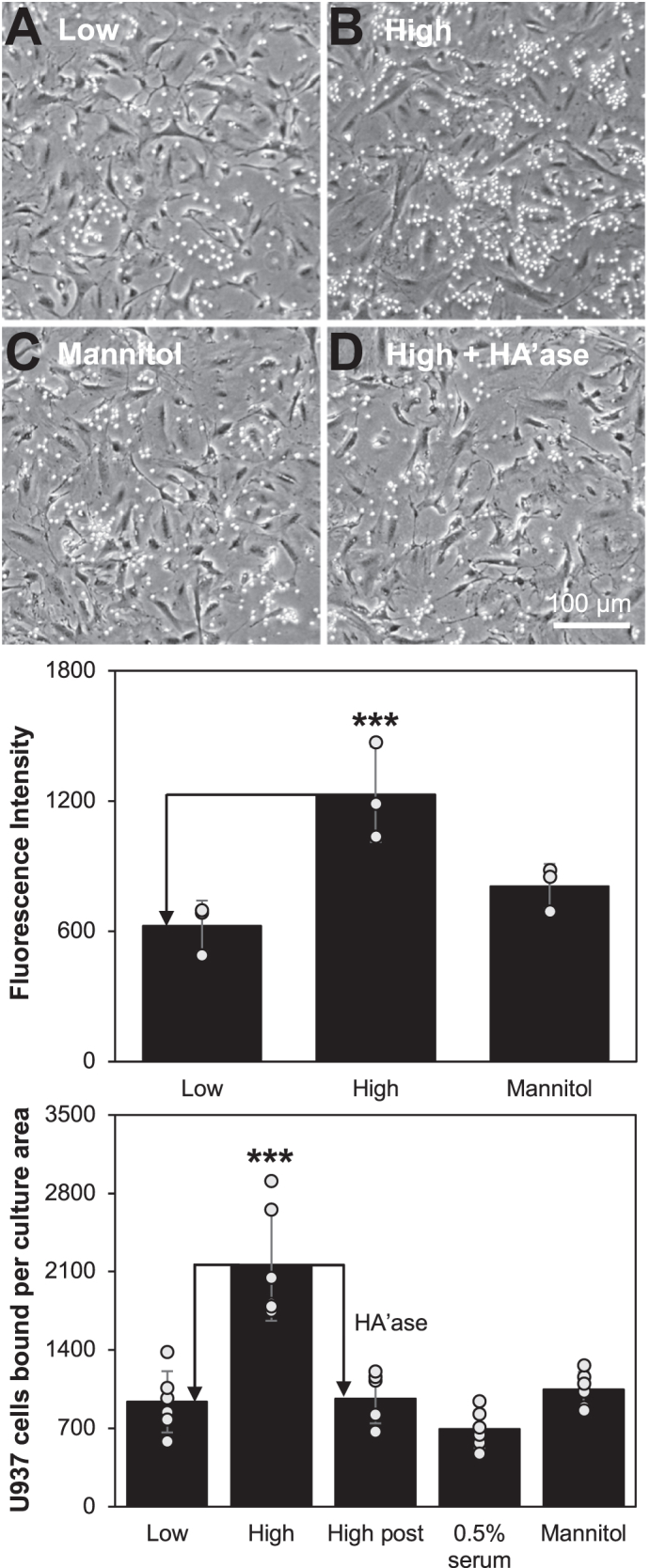
**Formation of monocyte adhesive HA matrix by SMCs.***Top panel*: Monocyte adhesion to airway SMCs using U937 cells. The serum-starved SMCs were incubated for 72 h with medium containing 10% FBS (*A*–*D*) in the presence of normal (5.6 mM, panel *A*) or high (25.6 mM, panels *B* and *D*) glucose. Mannitol (20 mM) with 5.6 mM glucose was used as an osmotic control (panel *C*). Hyaluronidase treatment (panel *D*) was used to determine the extent of HA-mediated monocyte adhesion to SMC cultures induced by the high glucose concentration. Middle panel: FACE analysis of HA contents in SMC cultures. Serum-starved SMCs in 6-well plates were stimulated with 10% FBS in the presence of 5.6 mM (Low), 25.6 mM glucose (High), and 20 mM mannitol with 5.6 mM glucose (Mannitol) for 72 h, and then HA contents were determined by FACE analyses. The mean values and SDs were calculated (∗∗∗*p* < 0.01; n = 4). *Bottom panel*: Quantitation of high glucose-dependent monocyte adhesion to SMCs. Serum-starved SMCs in 6-well plates were stimulated with 10% FBS in the presence of 5.6 mM, 25.6 mM glucose, and 20 mM mannitol with 5.6 mM glucose for 72 h, and then they were assayed for monocyte adhesion. The monocyte adherent cultures were further subjected to hyaluronidase (HA’ase) post-treatment. Low: 5.6 mM glucose; High: 25.6 mM glucose; Mannitol: 5.6 mM glucose + 20 mM mannitol; High post: 25.6 mM glucose + HA’ase; and 0.5% serum: SMCs culture 0.5% FBS with 25.6 mM glucose. The cell cultures were imaged by microscopy with a Polaroid digital camera, and the numbers of monocytes per culture area were counted using the Image J software. The mean values and SDs were calculated (∗∗∗*p* < 0.01; n = 6).

When the pre-confluent SMC cultures were incubated in 0.4% serum for 48 h, the starved cells were arrested in the G0/G1 phase as shown previously and subsequently re-enter the cell cycle after serum stimulation (40). In order to determine the formation of the monocyte adhesive HA matrix by SMCs in response to hyperglycemic glucose, serum-starved, near-confluent SMCs were cultured for 72 h in a medium with 10% FBS and normal (5.6 mM) or high (25.6 mM) glucose concentrations, or in 5.6 mM glucose with 20 mM mannitol. The monocyte interaction with cultured SMCs was assayed by monocyte adhesion using the U937 monocytic leukemia cell line as described in our previous studies (26, 41, 42, 43). The 25.6 mM glucose treatment of SMC cultures (Fig. 1 top panel: *B*) greatly increased monocyte adhesion to the cultures when compared with those to the cultures treated with 5.6 mM glucose (Fig. 1 top panel: *A*) or with 20 mM mannitol in 5.6 mM glucose as an osmotic control (Fig. 1 top panel: *C*). Most of the adhered monocytes to the cultures induced by high glucose were removed by treatment with testicular hyaluronidase after assessing monocyte adhesion (Fig. 1 top panel: *D*). This underscores the role of HA in the adhesion process. Further, the monocyte adhesion to SMCs cultured in high glucose with limiting serum (0.5%) was similar to ones in the 5.6 mM glucose cultures (data not shown), indicating that the presence of serum is required for the high glucose that induced the monocyte adhesion to the SMC cultures.

**Figure 2:**
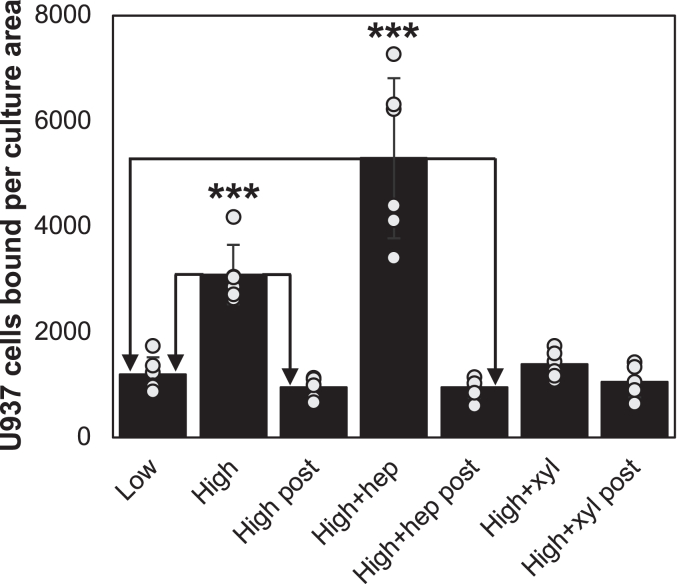
**Regulation of high glucose-dependent monocyte adhesion to SMCs by heparin and 4MU-xyl.** Serum-starved SMCs in 6-well plates were stimulated with 10% FBS in the presence of 5.6 mM, 25.6 mM glucose, and 25.6 mM glucose with or without 1 μg/ml heparin, or 0.25 mM 4MU-xyl for 72 h, and then assayed for monocyte adhesion. The monocyte adherent cultures were further subjected to hyaluronidase (HA’ase) post-treatment. Low: 5.6 mM glucose; High: 25.6 mM glucose; High post: 25.6 mM glucose + HA’ase; High+hep: 25.6 mM glucose + 1 μg/ml heparin; High+hep post: 25.6 mM glucose + 1 μg/ml heparin + HA’ase; High+xyl: 25.6 mM glucose + 0.25 mM 4MU-xyl; High+xyl post: 25.6 mM glucose + 0.25 mM 4MU-xyl+ HA’ase. The cell cultures were imaged by microscopy with a Polaroid digital camera, and the numbers of monocytes per culture area were counted using the Image J software. The mean values and SDs were calculated (∗∗∗*p* < 0.01; n = 6).

In separate experiments, in order to determine the HA synthesis by SMCs in response to hyperglycemic glucose, the HA contents of the cell layers were determined by FACE analysis as described under “Experimental procedures.” Figure 2 shows that the HA contents in SMCs incubated with 25.6 mM glucose were greatly increased compared with SMCs treated with 5.6 mM glucose or with 20 mM mannitol in 5.6 mM glucose as an osmotic control (Fig. 1 middle panel). Together, these results show that the high glucose treatment induced the formation of a monocyte adhesive HA matrix by SMCs, similar to the HA-mediated monocyte adhesion to poly I:C-treated smooth muscle cell cultures (26, 41, 43). These results were not present in SMC cultures incubated in normal glucose with or without mannitol as an osmotic control.

In the parallel experiments, in order to determine quantitatively the monocyte adhesion to SMCs in response to hyperglycemic glucose, triplicate serum starved, near-confluent SMC cultures were incubated for 72 h in medium with 10% FBS and normal (5.6 mM) or high (25.6 mM) glucose concentrations, or in 5.6 mM glucose with 20 mM mannitol, and then assessed for monocyte adhesion. The numbers of monocytes bound to the SMC cultures were then determined as described in our previous studies (23, 42). At 25.6 mM, there were ∼2.5 times as many adherent monocytes as observed with 5.6 mM glucose (Fig. 1 bottom panel), and almost all of the difference was diminished when SMC cultures exposed to 25.6 mM glucose were treated with hyaluronidase after assessing monocyte adhesion (Fig. 1 bottom panel: post). The mannitol osmolarity control showed no difference from the normal (5.6 mM) glucose cultures. Further, there is a correlation between increased HA content and increased adhesion of monocytes (Fig. 1 middle panel).

### Regulation of glucose-induced HA synthesis and monocyte adhesion in SMCs

Figure 2 shows the adhesion of U937 monocytes to SMC cultures at 72 h after the indicated treatments and quantification of the results is shown in the bar graphs. Significantly more monocytes bind to the cultures in hyperglycemic medium (high) than for cultures in medium with normal glucose (low). Hyperglycemic medium with 4MU-xyl, which diverts cytosolic UDP sugar substrates into the Golgi to synthesize extensive amounts of chondroitin sulfate (44), showed no increase in monocyte adhesion (high + 4MU-xyl). Further, there is no significant difference in HA contents in low glucose cultures and in high glucose cultures treated with 4MU-xyl. The ratios of HA contents from FACE analyses for 4MU-xyl high glucose cultures to the average values in the low glucose control cultures are 1.02 ± 0.27 *versus* 1.00 ± 0.19 (n = 3). In contrast, a hyperglycemic medium with heparin, which has been shown to prevent nephropathy and proteinurea in the streptozotocin diabetic rat (45, 46, 47), significantly increased monocyte adhesion above the cultures treated with hyperglycemia alone, as shown in the bar graphs (Fig. 2). This result is consistent with the results with mesangial cells (22). The increases in monocyte adhesion in both of these hyperglycemic treatments were prevented when the cultures were treated with hyaluronidase after assessing monocyte adhesion (Fig. 2), underscoring again the role of HA in mediating this monocyte adhesion.

### Effect of heparin concentration on monocyte adhesion to SMCs

**Figure 3:**
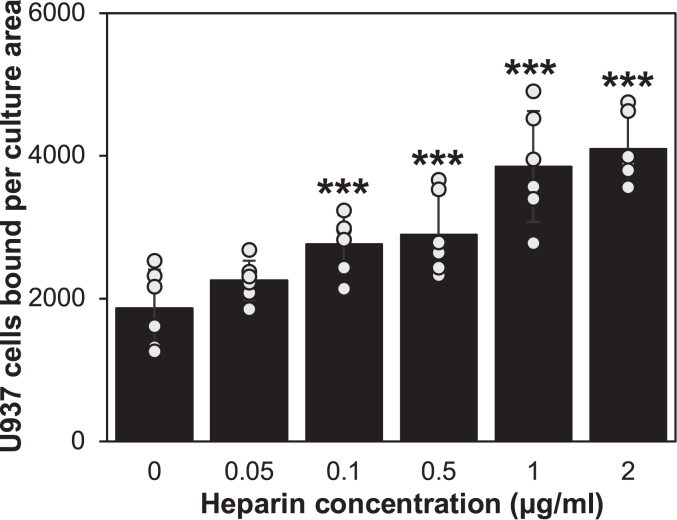
**Quantitation of dose-dependent monocyte adhesion induced by heparin to SMCs.** Serum-starved SMCs in 6-well plates were stimulated with 10% FBS in 5.6 mM glucose, 25.6 mM glucose, or 25.6 mM glucose in the presence of 0.05 to 2.0 μg/ml heparin for 72 h and then assayed for monocyte adhesion. The cell cultures were imaged by microscopy with a Polaroid digital camera, and the numbers of monocytes per culture area were counted using the Image J software. The mean values and SDs were calculated (∗∗∗*p* < 0.01; n = 6).

In the experiment in Figure 3, near-confluent and serum-starved SMC cultures were incubated for 72 h in a medium with 10% FBS and hyperglycemic (25.6 mM) glucose concentration, or in the presence or absence of 0.05 to 2 μg/ml heparin. Figure 3 compares the effects of different concentrations of heparin on U937 monocyte binding. Dose-dependent increases in monocyte adhesion induced by heparin were observed. The heparin treatments reached a plateau level ∼2 times the hyperglycemic medium alone at a concentration of 1.0 μg/ml heparin. A concentration of 0.10 to 0.5 μg/ml of heparin increased synthesis nearly midway to the plateau level. Previous studies including ours showed that there was heparin binding on G0/G1 SMCs with a K_d_ of 1.6 × 10^−8^ M (40). These results with low concentrations of heparin indicate that heparin acts on G0/G1 SMCs through a high-affinity interaction to regulate the HA matrix formation in hyperglycemic cultures.

**Figure 4:**
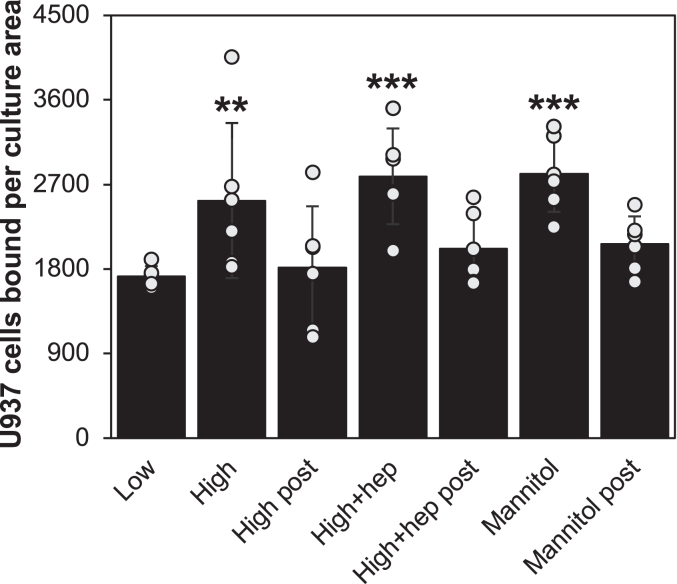
**The effect of confluency of SMC cultures on glucose-induced monocyte adhesion.** Confluent SMCs in 6-well plates were stimulated with 10% FBS in the presence of 5.6 mM (Low), 25.6 mM glucose (High), 25.6 mM glucose with 1 μg/ml heparin (High hep), and 20 mM mannitol with 5.6 mM glucose (Mannitol) for 72 h and then assayed for monocyte adhesion. The monocyte adherent cultures were further subjected to hyaluronidase (HA’ase) post-treatment. The cell cultures were imaged by microscopy with a Polaroid digital camera, and the numbers of monocytes per culture area were counted using the Image J software. The mean values and SDs were calculated (∗∗*p* < 0.05; n = 6) (∗∗∗*p* < 0.01; n = 6).

However, heparin’s high-affinity interaction on SMCs was observed on the G0/G1 SMC surface but not on the confluent cultures (40, 48). To determine to what extent hyperglycemic glucose and heparin-induced HA matrix formations depend on the SMC growth state, confluent non-dividing SMCs were treated for 72 h in medium with 10% FBS and normal (5.6 mM), or high (25.6 mM) glucose concentrations or 25 mM glucose in the presence of 1.0 μg/ml heparin, or 5.6 mM glucose with 20 mM mannitol and then assessed for monocyte adhesion (Fig. 4). The number of monocytes bound to the SMC cultures were then determined, and then the HA matrix that mediated monocyte adhesion was determined by hyaluronidase treatment after assessing monocyte adhesion. There were about 25% increases in SMC cultures induced by hyperglycemic glucose alone and in heparin-treated hyperglycemic glucose compared with the normal glucose cultures. Further this small increase in the monocyte adhesion was also observed in the culture treated with mannitol, suggesting that this response could be due to an osmotic effect. These data confirmed that the heparin’s high-affinity interaction on SMCs is not present on the confluent non-dividing SMC surface.

### Regulation of gene expression related to HA matrix synthesis in SMCs

**Figure 5:**
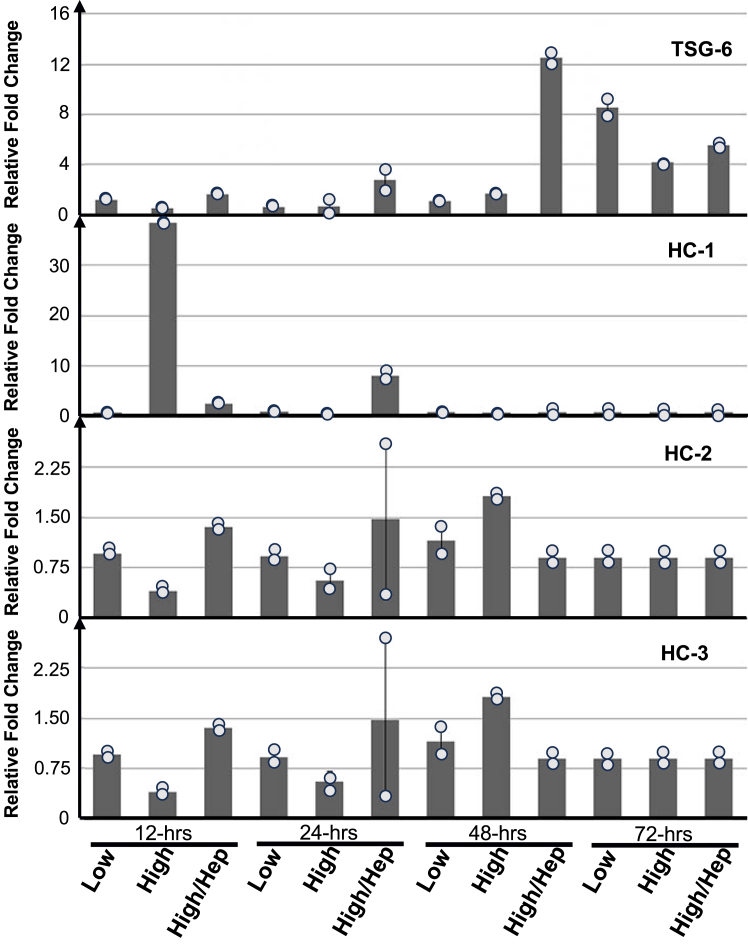
**Expression of Genes related to the formation of HA matrix in SMCs.** Serum-starved SMCs in 6-well plates were stimulated with 10% FBS in the presence of 5.6 mM, and 25.6 mM glucose with or without 1 μg/ml heparin for 12, 24, 48, and 72 h, and then the RNAs were extracted for RT-PCR analysis for TSG-6, HC 1, 2, and three expressions using the primers listed in Table 1 with beta-actin as a housekeeping gene for normalization. The data were expressed as a mean of two independent measurements.

Hyperglycemic glucose induced the formation of a monocyte adhesive HA matrix, and it has also been shown that the HC transfer from IαI to HA that is catalyzed by TSG-6, has an important role in the function of the extracellular HA matrix (30, 31, 49). However, it is still unknown to what extent hyperglycemic glucose induces the expression of genes that directly regulate the formation of this HA matrix. To address this issue, serum-starved, near-confluent SMCs were cultured for 12, 24, 36, 48, and 72 h in medium with 10% FBS and normal (5.6 mM) glucose concentration, or in high (25.6 mM) glucose concentration, or in high (25.6 mM) glucose concentration with 1 μg/ml heparin. At the end of each incubation time, the cells were harvested for real-time PCR analyses of TSG-6, HC 1, 2, and 3. The primers for the reactions are listed in Table 1 as described in our previous study (50). There were no significant changes in the expression of the HCs in all samples tested. However, there was increased expression of TSG-6 in the 72 h cultured samples with or without treatments (Fig. 5). Interestingly, in the heparin-treated samples, there were earlier induced expressions of TSG-6 observed in the 24 and 48 h samples, indicating that SMCs in culture do express TSG-6 at the time when the monocyte adhesive HA matrix forms. Since TSG-6 is one enzyme that can catalyze the HC transfer to HA structures, these results also suggest that the HA matrix might have been modified by HCs *via* covalent ester bonds.

**Table 1:** The sequences of the PCR amplification primers

Name of primers	Primer sequence	Expected PCR product
β-Actin	5′-GGTCATCACTATTGGCAACG-3′ 5′-ACGGATGTCAACGTCACACT-3′	133 bp
Heavy chain 1	5′-TTCTCAGCCCTTAGAGATGGTG-3′ 5′-GAGTGGCAACTTTGAGTCTATGG-3′	100 bp
Heavy chain 2	5′-GGTGATAGAGAATGATGCTGGA-3′ 5′-CAACCTTGGTGCCATAATACAG-3′	101 bp
Heavy chain 3	5′-CCCAGAAAGATTACAGGAAGGA-3′ 5′-TCGGTATGGACACCATCAATTA-3′	100 bp
TSG-6	5′-CTCCATATGGCTTGAACAAGCAGC-3′ 5′-ACCACCTTCAAATTCACATACG-3′	112 bp

### Confocal analysis of HC transfer on HA matrix in SMCs in response to hyperglycemic glucose with or without heparin

**Figure 6:**
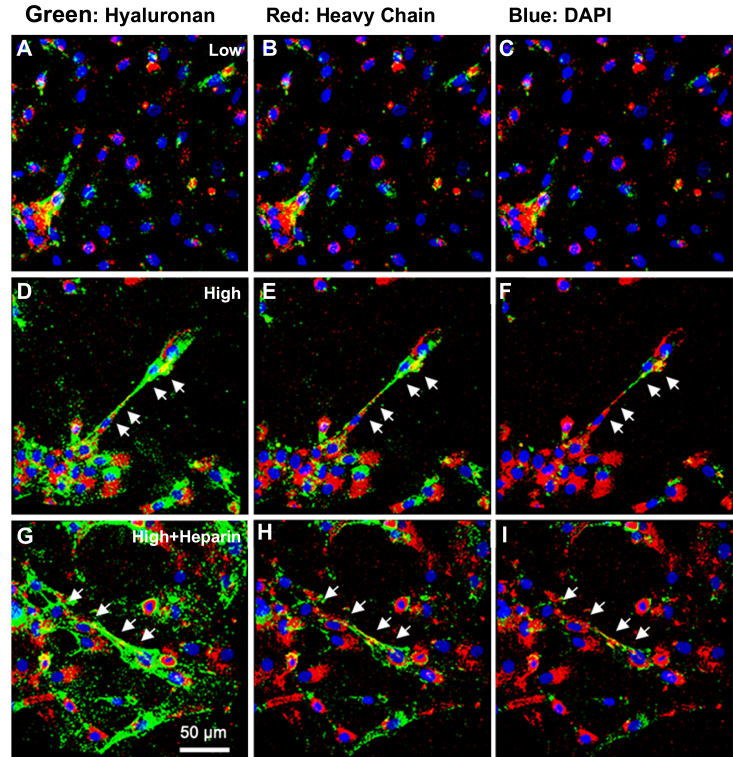
**Confocal microscopic analysis of HC transfer to HA matrix from IαI and pre-IαI in SMC cultures.** The serum-starved glomerular SMCs on chamber slides were incubated for 72 h with medium containing 7.5% FBS and 2.5% mouse serum in the presence of normal (5.6 mM, panels *A*–*C*) or high (25.6 mM, panels *D*–*F*) glucose, or high glucose + heparin (25.6 mM glucose and 1 μg/ml heparin, (panels *G*–*I*). HA was stained with HABP (*green*), HCs were stained with Dako antibody, and nuclei were stained with DAPI (*blue*). There are large HA structures co-stained with HCs in the cultures treated with high glucose (panels *D*–*F*) or with high glucose plus heparin (panels *G*–*I*) (*white arrows*).

In order to determine whether there was HC transfer on the extracellular HA matrix formed by the cultured SMCs, serum-starved, near confluent glomerular SMCs were cultured for 72 h in medium with 7.5% FBS and 2.5% mouse serum and normal (5.6 mM) or high (25.6 mM) glucose concentrations, or high (25.6 mM) glucose concentration with 1 μg/ml heparin. Then the cultured cells were stained for HA (green) and HCs (red) by HA-binding proteins and anti-IαI antibody (Fig. 6). There were large cable HA structures observed in the high glucose (Fig. 6, *D*–*F*, and in high glucose plus heparin treated cultures (Fig. 6, *G*–*I*), and these large HA cable structures were not found in the low glucose culture (Fig. 6, *A*–*C*). In order to observe the red staining closely, green staining signals were decreased in Figure 6, *B*, *C*, *E*, *F*, *H*, and *I*, and the red staining signals became more dominant. It is clear that there were red-stained coalesced HC structures (pointed by arrows) along the green HA structures. Interestingly, along the continuous HA cable structures, the red-stained coalesced HC structures were not forming the yellow signals, suggesting that red staining was not co-localized spatially with the green HA signals. These results suggest that the modification of HA structures by HC transfer was not occurring evenly along the cable structures, that is, there were some regions of HA cables with high heavy chain transfer while others were with much less transfer. In the high HC transfer regions, the dense HC attachment on the HA could block this part of the HA structures being accessed by the HA binding protein for the green HA staining signals, and in this case, there were no yellow signals observed. It is possible that these heavily transferred regions of HC modifications may have an important role in the pathological process. Further, these heavily transferred regions of HC modifications on HA structures were also observed in the heparin-treated cultures, suggesting that the presence of 1 μg/ml of heparin would not affect the HC transfer onto the HA structures.

### *In vitro* analysis of the effect of heparin on HC transfer to HA induced by TSG-6

The heavily transferred regions of HC modifications on HA structures were observed in both high glucose and high glucose plus heparin cultures, suggesting that heparin would not impede the TSG-6 catalyzed HC transfer to the HA structure. However, previous studies have shown that TSG-6 can crosslink HA *via* noncovalent molecular interactions that can be interfered by the presence of heparan sulfate *in vitro*. We have developed an *in vitro* assay for TSG-6-induced HC transfer to HA and used this method to determine to what extent the heparin affects the HC transfer to HA induced by TSG-6.

**Figure 7:**
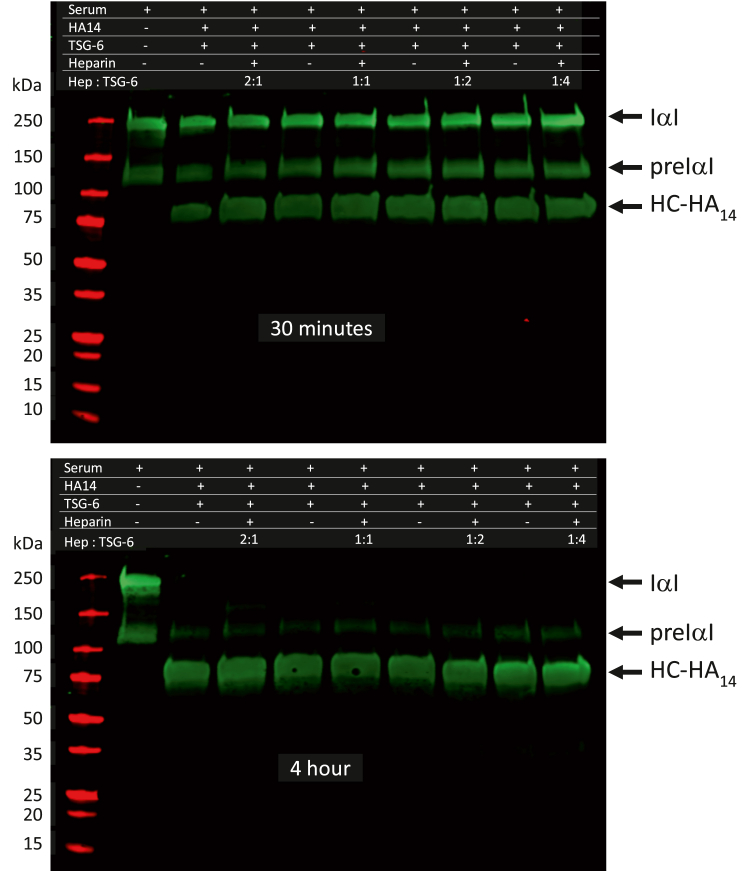
***In vitro* assay of effects of heparin on TSG-6 induced HC transfer to HA.** HA14 was incubated with human serum in the presence of different molar ratios of TSG-6 and heparin indicated on the blot at 37 °C for 0.5, 1, 2, and 4 h. At the end of incubation, reaction mixtures were subjected to Western analysis with the Dako IαI antibody. HC transfer to HA was markedly noticeable after a 0.5 h incubation (*upper panel*), reaching a steady state between 2 to 4 h (*lower panel*), and no inhibitory activities were observed upon the heparin treatment.

The *in vitro* assay of HC transfer used TSG-6 to induce the HC transfer from IαI in human serum to the HA14 oligosaccharide receptor. Production of HC-HA14 was quantified by Western blot analysis using an anti-IαI antibody that specifically recognizes the HC. The reactions were done in 25 μl PBS containing 1 mM MgCl2, 5% human serum, 1.25 μg HA14 oligosaccharide, and 3.5 pmol TSG-6, or in the presence or absence of 0.89, 1.74, 3.57 and 7.1 pmol heparin, and the ratios of heparin to TSG-6 were 2:1, 1:1, 0.5:1, and 0.25:1. The reaction mixtures were incubated at 37 °C for 30 min, and for 1, 2, and 4 h. At the end of incubation, 0.5 μl 0.5 M EDTA was added to stop the reaction. Then HC-HA14 contents were quantified by Western blots using anti-IαI antibody. Figure 7 shows the time course and dose dependency of heparin effects on the TSG-6-induced HC transfer by Western blot analysis. The reactions occurred rapidly with significant increases in HC-HA productions under all conditions observed in the 30 min (Fig. 7 upper panel). At the same time, the decreases in IαI and pre-IαI were observed, confirming that the HCs on the IαI and pre-IαI were transferred to the HA14 acceptor. By the end of 4 h incubation (Fig. 7 lower panel), almost all of the IαI and pre-IαI signals detected by their HCs had disappeared, and the HC-HA14 reached a plateau, indicating the completion of the reaction. There were no inhibitory effects of heparin on the TSG-6-induced HC transfer to the HA. In contrast, the presence of heparin slightly increases the HC transfer, which is clearly observed when the ratios of TGS-6 to heparin were 1:1, 1:2, and 1:4. Thus, the interaction of heparin with TSG-6 could be due to increases in either its binding to HA or its transferase activities, and at both circumstances, the TSG-6 induced formation of HC-HA complexes can be increased. Altogether, these studies indicate that heparin treatment will not impede the formation of the extracellular HA matrix.

### *In vitro* analysis of the effect of serum on HC transfer to HA induced by TSG-6

**Figure 8:**
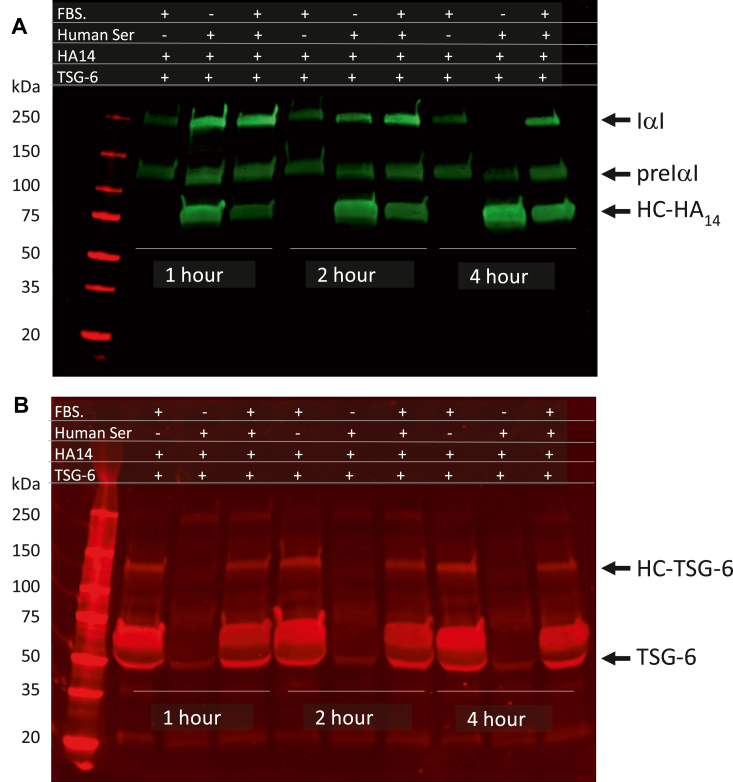
***In vitro* assay of the serum effects on HC transfer to HA.** HA14 and TSG-6 were incubated with FBS, human serum, or a mixture of FBS and human serum with 1:1 ratio at 37 °C for 1, 2, and 4 h. *A*, at the end of incubation, reaction mixtures were subjected to Western analysis with the Dako anti IαI antibody (*green*). HC transfer to HA in human serum alone was markedly noticeable after a 1 h incubation, reaching a steady state between 2 to 4 h, and there were not any transferring activities observed in FBS alone. However, there were inhibitory activities in human serum mediated HC transfer by FBS. *B*, the same blot of panel (*A*) was probed by anti-TSG-6 antibody (*red*).

The anti-IαI antibody to detect HCs from Dako has the reactivity to human and mouse serum, but fetal bovine serum (FBS) or calf serum have been used broadly in cultured studies. In order to determine the HC transfer in the cell cultures using FBS, small amounts of mouse serum were supplemented. However, it is still unknown to what extent the presence of FBS could affect the HC transfer from mouse serum IαI and pre-IαI to the HA matrix. Thus, an *in vitro* assay was used to determine the effect of the serum factor on the TSG-6-induced HC-HA formation (Fig. 8*A*). Surprisingly, although the Dako antibody does recognize the IαI and pre-IαI in the FBS, the HC-HA was not formed in the reaction mixture for all time points, suggesting that there were factors that impede the HC transfer from fetal bovine IαI and pre-IαI to HA. However, when human serum is used, the rapid transfers of the HC from human IαI and pre-IαI to HA were observed as expected. Interestingly, when the FBS was added, although the HC transfer was observed, the quantity of the HC-HA formation was to a lesser extent, suggesting that serum factors from FBS may slow the reaction. Altogether, these results indicate that using human or mouse serum as a supplement can efficiently detect the HC transfer to form HC-HA. Further, when the same blot was probed with the anti-TSG-6 antibody, the recombinant human TSG-6 was detected as expected as a single band at 50 kd in the reaction mixture with human serum alone (Fig. 8*B*). However, in the reaction mixtures with the presence of FBS, the TSG-6 was detected as a smear region with 55 to 70 kd, suggesting that the serum factors in FBS can alter the form of the complexes. It could be that these complexes of TSG-6 formed with serum factors in the FBS block the activity of TSG-6 in inducing the HC-HA formation when FBS is present in cultures.

## Discussion

In this study, we have determined the roles of hyperglycemia in inducing HA synthesis in dividing airway SMCs as well as to what extent heparin treatment impacts the high glucose-induced responses. Since extracellular HA matrix has important roles in both inflammatory and repairing processes, we determined to what extent this HA matrix has been modified by HC transfer from serum IαI and pre-IαI induced by either high glucose treatment and/or by high glucose plus heparin treatment. These studies provide new insights regarding the roles of HA matrix in mediating the pathophysiological process in hyperglycemia-induced lung injuries.

Under hyperglycemia, dividing SMCs stimulated from G0/G1 stage by FBS form the monocyte adhesive HA matrix in response to high glucose stress, which is not due to osmatic stress. Further, when the serum-starved G0/G1 SMCs and confluent SMCs were used, the high glucose fails to induce the monocyte adhesive matrix formation, probably due to that the serum-starved cells were growth arrested at G0/G1 phase, and confluent SMCs exhibit very minimal growth activity due to contact inhibition in culture. These results clearly suggest that cell growth and cell cycling events are required for this high glucose-induced monocyte adhesive HA matrix formation, consistent with our previous findings in the cultures of glomerular SMCs (mesangial cells) (42) and in the observations made by Evanko and Wight in the dividing SMCs cultured under a high glucose concentration (51).

In the normal tissues, the SMCs are in a quiescent state, and growth or proliferative activity is very minimal. A previous study (52) showed that, under this condition, an increase in the glucose-induced influx by elevated glucose transporter on the SMC surface and subsequent activation of glucose intermediate metabolic pathways will not promote the HA matrix synthesis, while there is an increase in HA synthesis under the same condition in the plaques where the SMC proliferation or growth activity is apparent. These *in vivo* studies are consistent with the findings in this study and in others (42, 51) that hyperglycemia-induced HA synthesis was observed in the dividing SMCs. The interaction of this SMC HA matrix with immune cells initiates inflammatory responses that lead to pathological changes in the vasculature (53).

Hyperglycemia activates the influx of glucose into the dividing cells leading to the elevation in the cytosolic substrates (UDP-glcUA and UDP-glcNAc), which initiates the intracellular synthesis of HA and deposits this high MW polyanionic glycosaminoglycan in intracellular compartments (endoplasmic reticulum (ER), transport vesicles, golgi) (22, 23, 24, 25). This initiates ER stress and unique autophagy with subsequent extrusion of an extracellular monocyte-adhesive HA matrix after the division that leads to inflammatory responses (22, 23, 24, 25). We propose that Mpi that are formed in high glucose are recruited into the diabetic tissues and initiate inflammatory and fibrotic responses that are ineffective in removing the HA matrix, which mediates the initiation and progression of diabetic complications.

Clowes and Karnovsky (54) in 1977 showed that the anti-mitogenic and/or anti-proliferative activities of heparin in smooth muscle cells could be independent of its well-known anti-coagulant activity. Since then, continuous efforts have been made to determine the roles of heparin in regulating the SMC growth response and matrix synthesis under various physiological and pathological conditions. Most of these studies used relatively high heparin concentrations above 100 μg/ml, and in this case, probably low-affinity interaction between heparin and SMCs in its biological regulations was investigated. However, several studies (48, 55, 56, 57), including ours (40), have shown that there are high-affinity heparin receptors on the serum-starved and growth-arrested smooth muscle cells and mesangial cells, suggesting that low concentrations of heparin would be sufficient to induce the HA matrix formation under hyperglycemia. Indeed, the dose-dependency of heparin studies shows a concentration of heparin as low as 0.01 μg/ml heparin can induce a significant increase in the HA matrix formation, and it reaches a plateau at 1 μg/ml, consistent with our finding in the mesangial cell cultures (39). These results suggest that a K_d_ of ∼20 nM would be the affinity binding that mediates heparin’s response, indicating that the heparin-induced HA response is *via* the high-affinity interaction between heparin and SMCs. Further, it is also known that a low-affinity binding of heparin was not observed in the confluent SMC cultures, which will predict that the effect of heparin on the induction of HA matrix is very mild. Our observation that heparin on confluent non-dividing SMCs shows mild effects supports this observation.

Heparin is a highly sulfated, hence a highly polyanionic, glycosaminoglycan with a repeating disaccharide that contains a hexuronic acid (either glucuronic acid or iduronic acid) and glucosamine (either N-acetylated or N-sulfated). Daily IP injections of a small amount of heparin in the Streptozotocin (STZ) diabetic rats prevented the pathological HA responses even though the animals sustained hyperglycemic levels of glucose throughout (45, 46). This led to clinical trials with heparin for the treatment of patients with diabetes (36). However, the molecular and cellular mechanism(s) underlying the roles of heparin are still unclear. As shown in our previous study (39), low concentrations (K_d_ ∼20 nM) of heparin prevent intracellular HA synthesis in hyperglycemic dividing cells and reprogram them to synthesize a monocyte-adhesive extracellular HA matrix after division (22). We propose that the tissue-repairing monocytes/macrophages (Mtr) under hyperglycemia treated with heparin effectively remove the HA matrix without initiating the inflammatory and fibrotic responses, which allows the cells and tissue to maintain their functions while still synthesizing a monocyte-adhesive HA matrix in response to the hyperglycemia. This prevents the cytosolic concentrations of UDP-GlcNAc and UDP-GlcUA from increasing to unacceptable levels during cell division under glucose stress. This study also indicates that heparin-induced robust formation of monocyte adhesive HA matrix by SMCs on the one hand prevents intracellular HA, ER stress, autophagy, and subsequent inflammatory responses, which sustains the SMC function under hyperglycemia. On the other hand, our previous study also showed that heparin treatment prevents Mpi formation and induces the Mtr phenotype that removes the SMC monocyte adhesive HA matrix (58). Together, these implicate the therapeutic roles of heparin and its related molecules in diabetes-induced lung injuries.

4MU-xyl, which has been used previously in preclinical studies (59, 60, 61), also inhibits stress functions by a mechanism different from heparin (24). The presence of 4MU-xyl prevents intracellular HA synthesis by entering the Golgi and greatly increasing chondroitin sulfate (CS) synthesis (22, 44). This requires transport of UDP-GalNAc and UDP-GlcUA from the cytosol into the Golgi (22, 24, 44, 62). Since UDP-GalNAc is derived by epimerization of UDP-GlcNAc, this prevents cytosolic concentrations of UDP-GlcNAc and UDP-GlcUA from increasing to unacceptable levels during cell division (62). Our observation with 4MU-xyl supports the notion that 4MU-xyl treatment lowers HA synthesis by diverting cytosolic UDP-GlcNAc and UDP-GlcUA to Golgi to synthesize the chondroitin sulfates that are secreted extracellularly, which provides another way to maintain cellular and tissue function by ameliorating the cytosolic high glucose stress.

Our previous studies and others have shown that modification of the HA matrix by HC transfer from IαI and pre-IαI to form HC-HA has important roles in the pathogenesis of various inflammatory diseases including rheumatoid arthritis and asthma. However, in the cell culture model with FBS in the growth medium, which is the main source of IαI and pre-IαI, the HC modification has hardly been seen with Dako antibody in some studies where the formation of a monocyte adhesive HA matrix was apparent (26). One possible explanation could be that the reactive specificity of this antibody was only for the human and mouse HCs and not for the bovine HCs. However, there is no experimental data to support this notion. This study shows that this antibody can recognize IαI and pre-IαI from both FBS and human serum and even detected signals in FBS that were weaker when the same concentration of serum was used. This indicates that either the reactivity of this antibody to bovine IαI and pre-IαI is less than one towards human species or the concentrations of IαI and pre-IαI in FBS were lower than in the human serum. Nevertheless, our *in vitro* assay showed that the HCs from human IαI and pre-IαI can be transferred to HA induced by TSG-6, which does not occur when FBS is used. These data provide evidence that there were serum factors present in the FBS that impeded the TSG-6-induced reaction. Indeed, when Western detection of TSG-6 was used for the reaction mixtures with FBS or human serum, TSG-6 was present as a single band run at the position of 50 kd, and there was a diffuse distribution of TSG-6 signal between 55 kd and 70 kd, suggesting that there were serum factors interacting with TSG-6, which interfered with the HC transfer to HA. Thus, results with FBS *in vitro* studies might not reflect what was happening *in vivo* in terms of the formation of the monocyte adhesive HA matrix.

There are two binding sites on TSG-6 for glycosaminoglycans, one for HA and another for the heparan sulfate or heparin. A recent study showed that the binding of TSG-6 to heparan sulfate or heparin affects the TSG-6-mediated crosslinking of HA (63), suggesting that the presence of the treatment of heparin could affect the function of TSG-6 in mediating the HA matrix formation. However, this study showed that heparin would not inhibit TSG-6 catalyzed HC transfer to HA to modify the HA matrix, clearly indicating that under the heparin treatment the HC modified HA extracellular matrix formation will be induced. This would facilitate the recognition of this matrix by reparative monocytes/macrophages and maintain the function of tissues and cells.

Another interesting observation in this study is that there is an uneven distribution of HC modification along the HA cable structure, that is, some areas with heavily red staining for HCs and others with minimal red and mainly green for HA staining. However, the roles of modification of the HA matrix by HC transfer and the distribution pattern of these modifications in the physio-pathological processes are still unknown. It could be involved in: (1) recognition and interaction by inflammatory cells, (2) recognition and interaction by reparative cells such as monocytes/macrophages, and (3) stability of the extracellular matrix. Further, a previous study (64) also suggested that the HC-modified HA matrix involves induction of reparative macrophages differentiated from monocytes. Our future studies will determine to what extent HC modification of HA affects the responses and interactions by inflammatory monocyte/macrophages or by reparative monocyte/macrophages.

## Experimental procedures

### Reagents

*Streptomyces* hyaluronidase, Streptococcal hyaluronidase, chondroitinase ABC, and HA14 oligosaccharide (14 monosaccharides in length) were from Seikagaku America Inc. Mouse serum (S7273) was from Sigma. Human serum was used as our source of IαI (donor 736; Equitech-Bio Inc). Whole serum was used, and IαI was not purified from the serum. A biotinylated HA-binding protein (product 385,911) was from EMD Chemicals, Gibbstown, NJ. Recombinant human TSG-6 (2104-TS) was from R&D Systems. The antibody against IαI (A0301) was purchased from Dako North America, Inc. Two goat polyclonal HC antibodies (raised against the mouse antigen) (sc-33944 and sc-21978) were from, Santa Cruz Biotechnology.

### Establishment of SMC cultures

Airway SMC cultures were established from isolated tracheas of 1- to 2-month-old BALB/c mice as described in our previous studies (26, 43, 44). Glomerular SMC (mesangial cell) cultures were established from isolated glomeruli and characterized as described in our studies (40, 65, 66). SMCs were used between passages 5 and 15. SMCs were cultured in RPMI 1640 medium containing 10% fetal bovine serum (FBS) and passaged at confluence by trypsinization for 5 min with a solution of 0.025% trypsin, 0.5 mM EDTA. To render cells quiescent (40, 67), cultures at 40% confluence (2 × 10^4^ cells/cm^2^) were washed with RPMI 1640 medium and placed in fresh medium containing 0.4% FBS for 48 h (yielding 70–80% confluent cultures).

### Assay for monocyte adhesion

SMCs in six-well plates were treated up to 72 h with 5 to 20% FBS and concentrations of 5.6 and 25.6 mM D-glucose. U937 cells were cultured in suspension in RPMI 1640 medium containing 5% FBS and passaged at a 1:5 ratio (2 × 10^5^ cells/ml) every 48 h (41). Assays for monocyte adhesion were done as described previously (41, 42). After washing, the cell cultures were imaged by microscopy with a Polaroid digital camera (42), and the numbers of monocytes per culture area were counted using Image-J software. Each culture was equally divided into four regions and a culture area for imaging was randomly picked in each region. S. hyaluronidase treatment (1 TRU/ml at 37 °C for 15 min) of SMCs after monocyte incubation was used to determine the extent of the HA-mediated adhesion.

### Fluorophore-assisted carbohydrate electrophoresis analysis of reducing saccharides

Cell cultures were incubated with proteinase K at 250 μg/ml in 0.1 M ammonium acetate, pH 7.0, for 3 h at 60 °C (42, 68, 69). The reaction was terminated by heating the samples at 95 °C for 3 to 5 min. Glycosaminoglycans were recovered by 75% ethanol precipitation at −20 °C overnight and subsequent centrifugation. The pellets were dissolved in 0.1 M ammonium acetate, pH 7.0, and incubated with S. hyaluronidase (50 milliunits/ml) and chondroitinase ABC at 2 units/ml overnight at 37 °C to generate disaccharides from HA and chondroitin/dermatan sulfate. The reaction was terminated by heating the samples at 95 °C for 3 to 5 min. The digests were dried by centrifugal evaporation in microtubes and then subjected to reductive amination with 2-aminoacridone as described (42, 68, 69). At the end of the incubation, the samples were each mixed with glycerol to 20% and 5 μl aliquots were then subjected to electrophoresis on Glyko Mono Composition gels with Mono Running buffer from ProZyme Inc. Running conditions were 500 V at 4 °C in a cold room for 1 h. Gels were imaged on an Ultra Lum *trans*-illuminator (365 nm). Images were captured with a Quantix cooled charge-coupled device camera from Roper Scientific/Photometrics and analyzed with the Image J software. The HA contents were quantified according to the integrated intensity of signal bands and then normalized with DNA contents in the samples.

### Immunohistochemistry

SMC cultures on chamber slides were fixed in 4% paraformaldehyde at 4 °C overnight and then stained for HA with biotinylated HA-binding protein at a 1:100 dilution (Seikagaku America), for IαI HCs with an IαI antibody at a 1:100 dilution (A0301, Dako North America, Inc), and for nuclei with DAPI, as described (23, 42, 70) or according to the manufacturer’s instruction. Samples were treated with biotinylated HA-binding protein and antibody, washed, and treated with fluorescein isothiocyanate/streptavidin at 1:500 dilution and donkey anti-rabbit IgG Cy3 antibodies at 1:400 dilution (Jackson ImmunoResearch). Stained samples were mounted in VectaShield containing DAPI (Vector Laboratories) for staining the nuclei of cells. Confocal images of the samples were obtained with a Leica TCS-NT laser scanning confocal microscope equipped with three lasers for excitation at 351-nm, 488-nm, and 561-nm wavelengths at the Cleveland Clinic Image Core. The same settings of the confocal microscope and laser scanning were used for both control and treated samples.

### Heavy chain transfer reactions

The reaction volumes were 25 μl of PBS containing 1 mM MgCl_2_, 5% serum supplemented with 1.25 μg of HA, and/or 0.005 μg TSG-6. TSG-6 was always added last and marked the beginning of the incubation period (time 0) (71, 72). EDTA was used to stop the reaction by adding 0.5 μl of a 0.5 M (pH 8.0) solution. In the dose-dependent experiments, different molar ratios of heparin and TSG-6 were added to the same reaction mixture and then incubated for 0.5, 1, 2 and 4 h. All experiments included negative and positive controls, as described for each figure. Most experiments included internal replicates (*i.e.*, incubations at different time points). For those that did not, they were repeated for confirmation at least one time.

### Western blot analysis

Samples were electrophoresed on 4 to 15% mini-PROTEAN TGX gels (Bio-Rad) and blotted using the Bio-Rad nitrocellulose and *Trans*-Blot Turbo System (71, 72). Samples of 25 μl with 1.25 μl of serum gave a strong HC signal on the blots with an IαI antibody (A0301, Dako North America, Inc; 1:8000 dilution). The molecular weight standard was purchased from Li-Cor (928-40000). The blots were blocked for 1 h with Li-Cor blocking buffer (927-40000; Li-Cor) and then probed with the IαI antibody (dilution 1:8000) in the blocking buffer with 0.1% Tween 20 for 1 h. The blots were washed 5× in PBS with 0.1% Tween 20 and probed with an IRDYE secondary antibody (Li-Cor; 926-32213) at 1:15,000 dilution in blocking buffer with 0.1% Tween 20 and 0.01% lauryl sulfate for 45 min. The blots were washed as described previously and imaged on an Odyssey Infrared Imaging System (Li-Cor).

### Quantitative reverse transcription PCR

RNA was isolated using the RNAeasy kit (74104; Qiagen), and cDNA was prepared using Superscript first-strand synthesis system (11904-018; Invitrogen) as described in our previous study (50). The primers used are listed in Table 1. The PCR conditions were one cycle at 94 °C for 3 min, 40 cycles of 95 °C for 30 s, 55 °C for 45 s, 72 °C for 1 min, followed by one cycle of 72 °C for 1 min. These conditions were used for all primers.

## Data availability

All data are contained within the article.

## Conflict of interest

The authors declare that they have no conflicts of interest with the contents of this article.
